# Association of oral care with periodontitis and glycemic control among US adults with diabetes

**DOI:** 10.1186/s12903-023-03580-0

**Published:** 2023-11-21

**Authors:** Yuqing Zhang, Suzanne G. Leveille, Sarah M. Camhi, Ling Shi

**Affiliations:** 1 Robert and Donna Manning College of Nursing and Health Sciences, University of Massachusetts Boston, 100 Morrissey Boulevard, Boston, MA 02125 USA; 2 College of Nursing, University of Cincinnati, 3110 Vine Street, Cincinnati, OH 45221 USA; 3 College of Arts and Sciences, Department of Kinesiology, University of San Francisco, Fulton Street, San Francisco, CA 94117 USA

**Keywords:** Diabetes mellitus, Oral health, Primary prevention, Blood glucose, Population health

## Abstract

**Background:**

Studies indicate that treating periodontitis may benefit glycemic control among people with diabetes. It is unclear whether oral self-care such as flossing may reduce risk for periodontitis and improve glycemic control among people with diabetes. The purpose of this study was to examine associations between oral care, specifically, flossing and preventive dental care, with periodontitis and glycemic control, among US dentate adults with diabetes.

**Methods:**

We analyzed data from the National Health and Nutrition Examination Survey 2011–2014 for 892 participants aged 30 years and older with diabetes who completed the periodontal examination and lab test for hemoglobin A1c (HbA1c). Sampling weights were applied. Multivariable logistic regression and multivariable linear modeling were performed to examine the associations of flossing and preventive dental services on periodontal health and HbA1c levels, respectively, controlling for sociodemographic characteristics, health behaviors, and other risk factors.

**Results:**

Among U.S. dentate adults with diabetes, 52.1% of flossers and 72.1% of non-flossers had periodontitis (*p* < 0.001). Flossers were 39% less likely to have periodontitis (Adj. OR 0.61, 95% CI 0.43–0.88) compared to non-flossers. Flossers had an average HbA1c reading 0.30% (95% CI 0.02%—0.58%) lower than non-flossers, adjusted for covariates (*p* = 0.037). Preventive dental visits were associated with reduced risk for periodontitis (Adj. OR 0.54, 95%CI, 0.38–0.75) but not glycemic control.

**Conclusion:**

Flossing was associated with periodontal health and glycemic control among US adults with diabetes. Although further research is needed, the findings support that oral self-care may be particularly beneficial for adults with diabetes.

**Supplementary Information:**

The online version contains supplementary material available at 10.1186/s12903-023-03580-0.

## Background

Diabetes mellitus and periodontal disease are two intertwined chronic diseases. US adults with poorly controlled diabetes have three times higher prevalence of severe periodontitis than those without diabetes [1]. Consequently, they lose approximately twice the number of teeth compared to their non-diabetic peers [2]. Periodontitis and the associated tooth loss can cause pain, distress and compromised nutritional intake, contributing negatively to quality of life and overall health. On the other hand, repeated and prolonged periodontal inflammation is known to mobilize the immune system to produce more pro-inflammatory cytokines, increase the whole-body inflammation load, and thereby induce insulin resistance [3, 4]. As a result, this bi-directional interaction between chronic periodontitis and diabetes mellitus jeopardizes glycemic control in persons with diabetes and increases risk for developing diabetic complications [5, 6].

Periodontitis is a chronic condition that is not cured but instead is managed. Oral self-care such as flossing and obtaining regular preventive dental care is essential for preventing periodontitis and for maintaining clinical efficacy after periodontal treatment [7, 8]. Both direct and indirect pathways might be involved in mechanisms linking periodontal inflammation and insulin resistance [9]. One widely cited explanation is that periodontal infection produces persistent low-grade inflammation in the oral cavity, induces increased secretion of host-derived inflammation biomarkers which exacerbate tissue breakdown, and eventually increase insulin resistance [9]. A number of studies have shown that interventions to reduce periodontal inflammation including non-surgical periodontal treatment such as scaling and tooth planing, reduces glycemic levels among people with diabetes, with reductions in HbA1 ranging from 0.27% to 1.03% [10]. In a randomized controlled trial among people with type 2 diabetes, non-surgical periodontal treatment reduced HbA1c by 0.9%, and also reduced serum levels of inflammatory biomarkers such as interleukins (IL-6, IL-12), and granulocyte colony-stimulating factor [11]. Given the association between periodontal inflammation and glycemic control [10, 11], it is plausible that regular flossing may also benefit glycemic control. Due to the lack of high-quality studies of oral self-care behaviors among people with diabetes, it is not clear whether oral self-care practices, such as flossing or regular preventive care, may enhance glycemic control among people with diabetes.

The purpose of this study was to examine the associations between oral health behaviors, specifically flossing and preventive dental care, and periodontitis and glycemic control, among US dentate adults with diabetes.

## Methods

### Study design

We performed a secondary data analysis of the National Health and Nutrition Examination Survey (NHANES) 2011–2014. NHANES is a continuous national health survey that is administered annually. We used data from the 2011–2014 waves because in 2011, NHANES began using an oral health questionnaire with additional questions pertaining to dental service utilization and, after 2014, the complex six-site periodontal examination protocol was discontinued. The NHANES employs a multistage probability-sampling mechanism to select about 5,000 participants each year and provides sampling weights to represent the US non-institutionalized civilian population [12]. More details about NHANES methodology are provided elsewhere [12]. NHANES data are publicly available and de-identified, therefore, this work was exempt from review by the Institutional Review Board of University of Massachusetts Boston.

### Participants

We included participants aged 30 years and older, self-reported as having diabetes, and having completed the NHANES home interview, the dental examinations, and bloodwork for HbA1c at the mobile examination center (MEC). Only people aged 30 years and older who had at least one natural tooth were eligible for the periodontal examination in the NHANES. In addition to excluding people who had missing data on key measures, we also excluded pregnant women because they could have gestational diabetes which is not the target of this study. In addition, we excluded people who were not eligible for the periodontal exam, specifically those who reported having a heart transplant, an artificial heart valve, congenital heart disease except mitral valve prolapse, or ever had bacterial endocarditis [13]. The final analytic sample included 892 participants aged 30 years and older with diabetes who completed the periodontal examination and laboratory testing for HbA1c (Supplementary Fig. 1).

### Measures

Sociodemographic measures were constructed based on participants’ responses to the NHANES survey questionnaires. Race/ethnicity was grouped as Hispanic, non-Hispanic White, non-Hispanic Black, and non-Hispanic Asian. Education level was grouped as follows: less than high school, high school graduate or equivalent, attended college or has an associate degree, or college graduate or above. Annual household income was grouped into four categories: < $20,000, $20,000–74,999, $75,000–99,999, and >  = $100,000. Health insurance coverage was based on health insurance through an employer or direct purchase as well as government programs like Medicare and Medicaid. Smoking status was derived from the smoking questionnaire, and classified as current smoking, former smoking, and never smoked. People who reported never having smoked at least 100 cigarettes in their lifetime were classified as never smoked. Body mass index (BMI) was calculated as weight in kilograms divided by height in meters squared, then grouped as follows: underweight (BMI < 18.5), normal weight (BMI 18.5 to 24.9), overweight (BMI 25.0 to 29.9) and obese (BMI 30.0 or higher).

Physical activity measurements were derived from the physical activity questionnaire. People who self-reported involvement in moderate or vigorous intensity recreational activities were further examined to see whether the national guidelines were met. People satisfying any of the following criteria were defined as meeting the goal of national guidelines [14, 15]: 1) total minutes of moderate intensity recreational activities of 150 min or more per week, 2) total minutes of vigorous intensity recreational activities of 75 min or more per week, or 3) not meeting either 1) or 2) but the sum of minutes spent on moderate and vigorous activities totaled 150 or more minutes.

Participants were asked questions in the NHANES survey about diabetes and oral health care practices. A positive case of diabetes mellitus was based on self-reported diagnosis of diabetes derived from the answer to the question, “*have you ever been told you have diabetes?*” We included only those who answered “yes” to this item (included type 1 and type 2 diabetes as NHANES does not differentiate types of diabetes). People who answered “borderline,” “refused,” or “don’t know,” or those with missing data were excluded from the analysis. We chose the self-reported diagnosis of diabetes because the central research focus of this study was oral self-care behaviors of people aware of their diagnosis of diabetes mellitus. In this paper, we refer to diabetes mellitus as diabetes throughout the article.

Glycemic control was based on the laboratory measure of HbA1c. The HbA1c measure was chosen over the fasting plasma glucose or postprandial glucose because HbA1c levels are less susceptible to fluctuations due to the short-term impact of diet and exercise [16]. Poor glycemic control was defined as a HbA1c reading of 8.0% or higher [17, 18].

The second outcome was periodontal health. We constructed a dichotomous periodontitis variable (Yes/No) for descriptive and regression analysis, according to the periodontitis definition by the US Centers for Disease Control and Prevention /American Academy of Periodontology (CDC/AAP) [19]. This classification was based on the combined consideration of measurements of probing pocket depth and attachment loss at the four interproximal sites in the NHANES exam [19]. A dentist performed the full-mouth periodontal examination including gingival recession and probing periodontium pocket depth on each natural tooth excluding the third molars, using an HU-Friedy periodontal probe [13].

For preventive oral self-care behaviors, the primary exposure of interest in this study was flossing behavior. However, we also included the utilization of preventive dental services as a secondary exposure of interest. The frequency of flossing was measured in NHANES by asking, “*Aside from brushing your teeth with a toothbrush, in the last seven days, how many days did you use dental floss or any other device to clean between your teeth?”* A dichotomous flosser/non-flosser variable was constructed using participants’ response to the above question as a proxy question; people reporting flossing at least one day in the past seven days were classified as flossers and others, as non-flossers. The utilization of preventive dental services in the past year was defined by combining answers to two questions from the oral health interviews. First, participants were asked, *“About how long has it been since you last visited a dentist?”* and second, they were asked the main reason for the visit. If they had a visit in the past year for preventive dental care which included a regular dental check-up or cleaning, or were called back following a check-up or cleaning, then they were classified as having had a preventive dental visit in the past year. Participants who selected the response option that they visited a dentist “for treatment of a condition that a dentist discovered at an earlier check-up or examination”, were also classified as having obtained a preventive dental visit in the past year because this response option indicates an earlier preventive dental service was performed. Those who answered, “something was wrong, bothering or hurting”, “don’t know”, or refused to answer, were classified as not having had a preventive dental visit in the past year.

### Statistical analysis

We applied NHANES sampling weights to adjust for over-sampling of racial/ethnic minority groups, adults aged 65 and older, low-income people, and for nonresponse to the home interview or medical examination in NHANES 2011–2014 survey [12]. Both the primary sampling unit variable and the pseudo-stratum variable were applied to the analysis using the SAS *surveyfreq* procedure (SAS Institute Inc., Cary, NC). Survey-weighted descriptive statistics were used to present sample characteristics according to the prevalence of periodontitis. We used chi-squared tests to compare the prevalence of periodontitis and poor glycemic control according to flossing status and use of preventive dental services. Multivariable logistic regression models were performed to examine the association of flossing on periodontal health, adjusting for potential confounders described above. Multivariable linear models were performed to examine the association of flossing with the outcome, glycemic control. Multicollinearity between the covariates was evaluated. Model assumptions and fit were examined and tested, respectively.

Covariates that potentially could confound the relationship between flossing and periodontitis and HbA1c were selected based on biological plausibility and accumulated findings from prior research and included in the modeling [20–22]. Sociodemographic characteristics (age, sex, race/ethnicity, education, income, and health insurance) often confound associations with health behaviors, health services utilization, and chronic diseases such as periodontitis and diabetes. Health risk factors such as smoking, BMI, and physical activity were included because they are not only associated with glucose control, but also reflect a person’s health consciousness which may be related to oral self-care practices.

All analyses used a hypothesis test with a 2-sided significance level of 0.05 and were conducted using SAS® version 9.4 (SAS Institute Inc., Cary, NC).

## Results

Based on the NHANES 2011–2014 sample weights, the 892 participants who self-reported having diabetes in this study were estimated to represent 29,544,610 adults with diabetes in the U.S. The weighted mean age of the study population was 60 years, 48.8% were women and 59.4% of the population was non-Hispanic White, 19.7% Hispanic, 15.4% non-Hispanic Black and 5.5% Asian (Table 1). In terms of education, the weighted percentage who did not complete high school, were high school graduates, attended college, or were college graduates, was 20.8%, 25.1%, 32.0%, and 22.1%, respectively. About 20.5% of the population had an annual household income below $20,000; and most of the population (87.3%) had health insurance The weighted prevalence of mild-to-moderate periodontitis and severe periodontitis among the adult population with diabetes was 48.0% and 10.2%, respectively (Table 1).

**Table 1:** Weighted sociodemographic characteristics, US dentate adults aged 30 years and over with diabetes, NHANES 2011–2014 (*N* = 892)

Characteristics	Total, N (weighted^a^ %)	No Periodontitis, n (weighted^a^ %)	Mild-Moderate Periodontitis, n (weighted^a^ %)	Severe Periodontitis, n (weighted^a^ %)	*P* Value^b^
**All**	892 (100)	308 (41.8)	462 (48.0)	122 (10.2)	NA
**Age**					
Mean (SE), in years	60.0 (0.4)	57.7 (0.7)	61.8 (0.5)	58.5 (1.0)	< .001^c^
**Sex**					
Male	456 (51.2)	126 (45.0)	250 (53.4)	80 (65.8)	< .001
Female	436 (48.8)	182 (55.0)	212 (46.6)	42 (34.2)	
**Race/ethnicity**					
Non-Hispanic White	278 (59.4)	121 (69.3)	137 (55.0)	20 (40.3)	< .001
Non-Hispanic Black	269 (15.4)	80 (12.5)	140 (15.6)	49 (25.7)	
Non-Hispanic Asian	100 (5.5)	33 (4.5)	51 (6.1)	16 (7.1)	
Hispanic	245 (19.7)	74 (13.6)	134 (23.4)	37 (27.0)	
**Education**					
< High school diploma	267 (20.8)	61 (13.1)	162 (25.1)	44 (32.4)	< .001
High school graduate/GED or equivalent	206 (25.1)	67 (25.1)	105 (24.9)	34 (26.2)	
Attend college or AA	248 (32.0)	97 (31.4)	128 (33.8)	23 (26.2)	
College graduate or above	169 (22.1)	83 (30.5)	65 (16.2)	21(15.2)	
**Annual income, $**					
< 20,000	234 (20.5)	68 (16.8)	128 (22.8)	38 (24.7)	< .001^d^
20,000–74,999	462 (55.7)	149 (51.7)	246 (58.2)	67 (61.1)	
75,000–99,999	61 (9.3)	27 (11.5)	26 (8.5)	8 (3.6)	
≥ 100,000	92 (14.5)	52 (20.0)	36 (10.5)	4 (10.5)	
**Health insurance**					
Yes	752 (87.3)	267 (88.9)	395 (88.8)	90 (74.0)	.002
No	140 (12.7)	41 (11.1)	67 (11.2)	32 (26.0)	

*Abbreviations*: *AA* Associate of Arts degree, *GED* General educational development, *NA* Not applicable

^a^Sampling weights were applied to generate US population estimates; groups were compared using the $\chi$ ^2^ test based on unweighted data

^b^*P* values calculated by Pearson $\chi$ ^2^ test

^c^*P* values calculated by ANOVA test

^d^*P* values calculated by Fisher’s Exact test

People who flossed, compared to those who never flossed were relatively younger (mean 59.4 vs. 61.2, *p* < 0.05), have a college education (% of college graduates 26% vs. 13.3%, *p* < 0.001) and less likely to have low-income (% annual income < $20,000: 17.8% vs. 26.6%, *p* < 0.001), respectively. The weighted prevalence of flossing was 69.5% (Table 2). The practice of preventive oral self-care was associated with lower prevalence of periodontitis and lower prevalence of poor glycemic control (HbA1c ≥ 8.0%) among US adults with diabetes. Specifically, the prevalence of periodontitis was significantly lower among people who practiced flossing than those who reported never flossing (52.1% vs. 72.1%, respectively, *p* < 0.001) (Table 3). The prevalence of periodontitis was also significantly lower among people who obtained preventive dental services compared to those who did not use preventive dental services (49.0% vs. 66.5%, respectively, *p* < 0.001). Similarly, the weighted prevalence of poor glycemic control was significantly lower among flossers than non-flossers (29.1% vs. 39.6%, respectively, *p* < 0.01); and it was also significantly lower among people who used preventive dental services compared with those who did not, (28.7% vs. 34.2%, respectively, *p* < 0.05).

**Table 2:** Weighted sociodemographic characteristics by flossing, US dentate adults aged 30 years and older with diabetes, NHANES 2011–2014 (*N* = 892^d^)

Characteristics	Total, N (weighted^a^ %)	Practiced Flossing, n (weighted^a^ %)		*P-* value^b^
		**No**	**Yes**	
**All**	892 (100)	310 (30.5%)	582 (69.5)	NA
**Age**				
Mean (SE), in years	60.0 (0.4)	61.2 (0.7)	59.4 (0.7)	< .05^c^
**Sex**				
Male	456 (51.2)	168(54.1)	288 (49.9)	0.189
Female	436 (48.8)	142(45.9)	294 (50.1)	
**Race/ethnicity**				
Non-Hispanic White	278 (59.4)	87 (55.1)	191(61.3)	0.495
Non-Hispanic Black	269 (15.4)	95 (16.7)	174 (14.8)	
Non-Hispanic Asian	100 (5.5)	38 (6.8)	62 (5.0)	
Hispanic	245 (19.7)	90 (21.4)	155 (18.9)	
**Education**				
< High school diploma	267 (20.8)	133 (30.8)	134 (16.4)	< .001
High school graduate/GED or equivalent	206 (25.1)	69 (25.8)	137 (24.8)	
Attend college or AA	248 (32.0)	78 (30.1)	170 (32.8)	
College graduate or above	169 (22.1)	29 (13.3)	140 (26.0)	
**Annual income, $**				
< 20,000	234 (20.5)	105 (26.6)	129 (17.8)	< .001
20,000–74,999	462 (55.7)	157 (53.5)	305 (56.7)	
75,000–99,999	61 (9.3)	11(6.9)	50 (10.3)	
≥ 100,000	92 (14.5)	23 (13.0)	69 (15.2)	
**Health insurance**				
Yes	752 (87.3)	253 (85.0)	499 (88.3)	0.107
No	140 (12.7)	57 (15.0)	83 (11.7)	
**Smoking Status**				
Never smoke	478(51.6)	159 (51.6)	319 (51.6)	0.359
Ex-smoker	292 (34.8)	102 (31.5)	190 (36.2)	
Current smoker	122 (13.6)	49 (16.9)	73 (12.2)	
**Weight Status**				
Under/Normal Weight	124 (12.0)	50 (15.7)	74 (10.4)	0.322
Overweight	266 (27.3)	88 (23.1)	178 (29.20	
Obese	491 (60.7)	165 (61.2)	326 (60.5)	

*Abbreviations*: *AA* Associate of Arts degree, *GED *General educational development, *NA *Not applicable

^a^Sampling weights were applied to generate US population estimates; groups were compared using the $\chi$ ^2^ test based on unweighted data

^b^*P* values calculated by Pearson $\chi$ ^2^ test

^c^*P* values calculated by ANOVA test

^d^Most categories has no missing data, the categories have missing data are education [*n* = 2], weight status [*n* = 11], and income [*n* = 43]

**Table 3:** Weighted prevalence of periodontitis and poor glycemic control according to practice of preventive oral self-care, US dentate adults with diabetes aged 30 and older, NHANES 2011–2014 (*N* = 892)

Oral Self Care Behaviors	Periodontitis^a^, n (weighted%)	*P*- value*	Poor glycemic control^b^ n (weighted %)	*P*- value^c^
**Use preventive dental service in the past one year**				
Yes	210 (49.0)	< .001	91 (28.7)	.007
No	365 (66.5)		162 (34.2)	
**Practices flossing**				
Yes	346 (52.1)	< .001	155 (29.1)	.033
No	238 (72.1)		103 (37.6)	

^a^Periodontitis includes all cases of periodontitis (mild, moderate, severe) according to CDC/AAP periodontitis definition

^b^Poor glycemic control defined as HbA1c of 8.0% or higher

^c^*P* values calculated on unweighted data by Pearson $\chi$ ^2^ test

The protective effect of flossing and use of preventive dental services on periodontitis persisted after controlling for potential confounders including sociodemographic factors (age, sex, education, income, race/ethnicity, and health insurance), BMI, smoking status, and HbA1c. Specifically, flossers were 39% less likely than non-flossers to have periodontitis (Adj. OR 0.61; 95%CI, 0.43–0.88) and people who used preventive dental services were 46% less likely than others to have periodontitis (Adj. OR 0.54; 95%CI, 0.38–0.75) (Table 4). Likewise, flossing was significantly associated with a HbA1c reading that was 0.3% lower than non-flossers, adjusted for covariates (Beta = -0.3, 95% CI -0.58, -0.02, *p* = 0.037). However, the use of preventive dental services was not significantly associated with HbA1c levels after controlling for covariates (Beta = -0.15, 95% CI -0.42, 0.13, *p* = 0.298).

In considering possible effect of multicollinearity, the variance inflation factor (VIF) was less than 2.5, indicating no multicollinearity existed in the HbA1c model. The adjusted model presented a better model fit than the unadjusted model measured based on the Akaike Information Criterion (AIC). Normality of the linear model was tested, and the HbA1C data were found to be slightly skewed to the right. In population studies, it is common to see skewness in biometric data. Thus, we used log transformation to adjust the skewness and improved model fit. In the adjusted *log transformed* model, flossing was significantly associated with a HbA1c reading that was 0.96% lower than non-flossers, adjusted for covariates (Beta = -0.96, 95% CI -0.99, -0.93, *p* = 0.0198). Therefore, the more conservative results derived from non-transformed model were presented (Table 3).

**Table 4:** Association among practice of oral self-care behaviors, periodontitis and glycemic control (HbA1c levels), US dentate adults with diabetes aged 30 and older, NHANES 2011–2014 (*N* = 892)

Predictors: Oral self-care behaviors	Outcome^a^: Periodontitis^b^						Outcome^c^: HbA1c					
	**Unadjusted model**			**Adjusted Model**			**Unadjusted Model**			**Adjusted Model**		
	**OR**	**95%CI**	***P* value**^**d**^	**AOR**	**95%CI**	***P* value**^**d**^	**Beta (95%CI)**	**SEB**	***P* value**	**Beta**	**SEB**	***P* value**
**Use preventive dental services in the past year**^**e**^												
No	1	[Ref]	< .001	1	[Ref]	< .001	[Ref]	–	.052	[Ref]	–	.298
Yes	0.45	0.34–0.60		0.54	0.38–0.75		-0.25 (-0.51,0.002)	0.13		-0.15 (-0.42,0.13)	0.14	
**Flossing behaviors**												
Non-flosser	1	[Ref]	< .001	1	[Ref]	.009	[Ref]	–	.016	[Ref]	–	.037
Flosser	0.45	0.33–0.62		0.61	0.43–0.88		-0.33 (-0.59, 0.06)	0.13		-0.30 (-0.58, -0.02)	0.14	

*Abbreviations*: *AOR* Adjusted odds ratio, *CI* Confidence interval, *Ref* Reference

^a^Logistic regression model adjusted for sociodemographic factors (age, sex, education, income, race/ethnicity, health insurance), body mass index, smoking status, and HbA1c. The magnitude of adjusted odds ratios derived from models in different studies or with different specifications should not be compared directly

^b^Periodontitis includes all cases of periodontitis (mild, moderate, severe) according to CDC/AAP definition

^c^Coefficients from multivariable linear regression model adjusted for sociodemographic factors (age, sex, education, income, race/ethnicity, health insurance), body mass index, being physically active, taking diabetic medication, smoking status, receipt of periodontal treatment, and presence of other systemic diseases including cardiovascular disease, cerebrovascular disease and lung disease

^d^*P* values calculated by the Wald test

^e^There are 13 missing data in preventive dental service

## Discussion

Using data from the 2011–2014 NHANES, our study results showed that well over half (58%) of US adults with diabetes had periodontitis, a figure much higher than the 42% prevalence of periodontitis in the general population of US adults, also reported from previous NHANES research [23]. Notably, we found the practice of preventive oral self-care behaviors (i.e. flossing and utilization of preventive dental services) was associated with better periodontal health in adults with diabetes. However, flossing but not obtaining of preventive dental visits was associated with reduced risk for poor glycemic control, adjusting for other factors. Overall, our results support the importance of preventive dental care especially routine flossing for periodontal health and glycemic control among people with diabetes.

Our findings are somewhat consistent with the work of Cepeda and colleagues, showing that among US adults (with or without diabetes), flossers are 23% less likely to have periodontitis than non-flossers [22]. However, we found a 39% reduced likelihood for periodontitis among the diabetes population that flossed regularly, indicating that adults with diabetes may experience even greater benefits than their non-diabetic peers in terms of periodontal health through practicing regular flossing.

The hypothesized mechanism underlying this research is that periodontal treatment reduces the local periodontium inflammation as well as systemic inflammation, measured by pro-inflammatory biomarkers. This decrease in systemic inflammatory burden contributes to reduced insulin resistance among people with diabetes [9, 24, 25]. Similarly, regular practice of preventive oral self-care helps to reduce local gum inflammation and would possibly benefit insulin resistance by lowering levels of systemic inflammation. Due to our cross-sectional design and the lack of biomarker measures in NHANES 2011–2014, we were not able to evaluate whether the relationship between flossing and glycemic control potentially is related to a reduction of systemic inflammatory burden. A pathway analysis using NHANES 2009–2010 data provided limited evidence to support this hypothesis [26]. Luo and colleagues reported flossing mitigates the effect of poor oral health on systemic inflammation as measured by C-reactive protein [26]. As others have observed, even very minor changes in HbA1c can have a major impact on clinical outcomes in diabetes [16]. Additionally, a large observational study reported that a 1-point (1%) reduction in the HbA1c level reduces risk by 12% for stroke, 21% for deaths related to diabetes, 14% for myocardial infarction, 19% for cataract extraction, and 43% for amputation [27]. In the present study, we found after adjusting for potential confounders, flossing was associated with 0.3% reduction in HbA1c. Although the magnitude is modest, this warrants further investigation using an experimental approach as it could be clinically meaningful in glycemic control among people with diabetes.

Periodontal disease is considered the sixth complication of diabetes mellitus [28]. Although it may not be considered as directly life-threatening as other diabetic complications, it influences glycemic control and systemic inflammation among people with diabetes. Therefore, the American Diabetes Association (ADA) recommends people with diabetes brush twice a day, floss once a day, and visit dentists twice a year [29]. However, despite the importance of brushing and flossing on periodontal disease prevention and management, people with diabetes are less likely to obtain preventive dental service and practice flossing than their counterparts without diabetes [30]. Moreover, there are missed opportunities for educating patients with diabetes about the proper technique of tooth brushing and flossing. A previous study of diabetes self-management education programs recognized by the ADA showed only about 10% of diabetes self-management curricula included in their survey have included the demonstration of proper tooth brushing/flossing techniques, and almost none of these programs had their clients demonstrate the recommended tooth brushing/flossing technique [31]. Substantial efforts are needed to improve diabetes self-management related to oral health care.

Obtaining preventive dental care and practicing effective toothbrushing and flossing are key oral health self-management behaviors and are particularly important for the population with diabetes. Understanding the nuance in the determinants and outcomes of different oral self-care behaviors is important to inform the design of oral self-care interventions and policy making. For example, we reported previously that having health insurance (as a proxy for dental insurance) is only associated with obtaining preventive dental visits but not with the practice of regular flossing [32], indicating that merely increasing dental insurance coverage may not resolve the issue of inadequate oral hygiene among people with diabetes. In this study, we found although both obtaining preventive dental visits and flossing are associated with lower risk for periodontitis among the diabetic population, only flossing was associated with reduced HbA1c after controlling for confounders. It is plausible that regular flossing works continuously against the daily accumulation of dental biofilm [33], which provides the source for local inflammation. Our results support the idea that obtaining regular preventive dental services cannot take the place of regular at-home flossing for the maintenance of oral health; both are important components in preventing onset or progression of periodontal disease, a highly prevalent diabetes complication. Moreover, diligent at-home oral hygiene maintenance, specifically, regular flossing may go further to reduce systemic inflammation, therefore benefitting daily glycemic control among people with diabetes. Our results are an important addition to evidence supporting that oral self-care behaviors be highlighted as a special component of diabetes self-management.

We recognize there are caveats of the study due to the limitations of the data. As with all cross-sectional studies, we cannot confirm the directionality or temporality of the associations that were found in this study. Secondly, we restricted our definition of oral self-care to flossing because NHANES does not provide data on brushing frequency among adults. Thus, we cannot determine the role tooth brushing may play in oral care and periodontitis prevention and glycemic control. Of note, we used the term, “flossing”, in reference to the question, “using dental floss or any other device”, which is a conventional approach in previous analyses using NHANES [22, 26]. Thirdly, key measures such as diabetes diagnosis, flossing, and the use of preventive dental visits were based on self-report and are susceptible to recall or reporting bias. Fourthly, the model construction was limited by the measurements available in NHANES dataset. The results could be potentially biased by unmeasured confounding, for example, health consciousness and intrinsic care-seeking behavior by participants might have been associated with both oral self-care behaviors and glycemic control and periodontal health. However, we partially control the influence by such unmeasured variables by controlling for socioeconomic factors (education, insurance, etc.,) and health risk factors (smoking, obesity, physical activities) instead of health consciousness. Results from observational studies are often limited by unmeasured confounding and only can be confirmed in randomized controlled trials whenever possible. Lastly, the magnitude of the association between flossing and glycemic control might be influenced by the right skew of the Hb1Ac distribution, however, by comparing the models before and after log transformation of the HbA1c levels, we recognized this modest skewness contributed to a conservative bias in the data we presented.

To the best of our knowledge, this is the first study to examine the association between good oral care and glycemic control with a focus on the role of oral self-care behaviors including flossing and preventive dental visits. This is a departure from previous studies that have focused on periodontal treatment [10, 34, 35]. The use of a nationally representative sample with use of sample weights in the analysis make the findings from of this study generalizable to the adult US population with diabetes.

In summary, we found flossing is associated with lower likelihood of periodontitis and better glycemic control among US adults with diabetes. In addition, preventive dental visits are associated with lower likelihood of periodontitis but not better glycemic control. This study highlights the importance of promoting preventive oral self-care, especially at-home oral hygiene behaviors, as an integral part of diabetes self-management. However, further longitudinal or experimental studies are warranted in order to advance oral health in persons with diabetes.

### Supplementary Information

**Additional file 1:** **Supplemental Figure 1. **Flow diagram for inclusion/exclusion to form the final analytical datasets.

## Data Availability

We performed a secondary analysis of data sets from the National Health and Nutrition Examination Survey 2022–2014. All data used in our study were publicly available were downloadable from the National Center for Health Statistics’ website. https://wwwn.cdc.gov/nchs/nhanes/Default.aspx.
