# Supplementary material for: Association of oral care with periodontitis and glycemic control among US adults with diabetes
Source: BMC Oral Health. 2023 Nov 21;23:903. doi: 10.1186/s12903-023-03580-0 (PMC10664594; doi:10.1186/s12903-023-03580-0)

**Supplemental Figure 1. Flow diagram for inclusion/exclusion to form the final analytical datasets.**


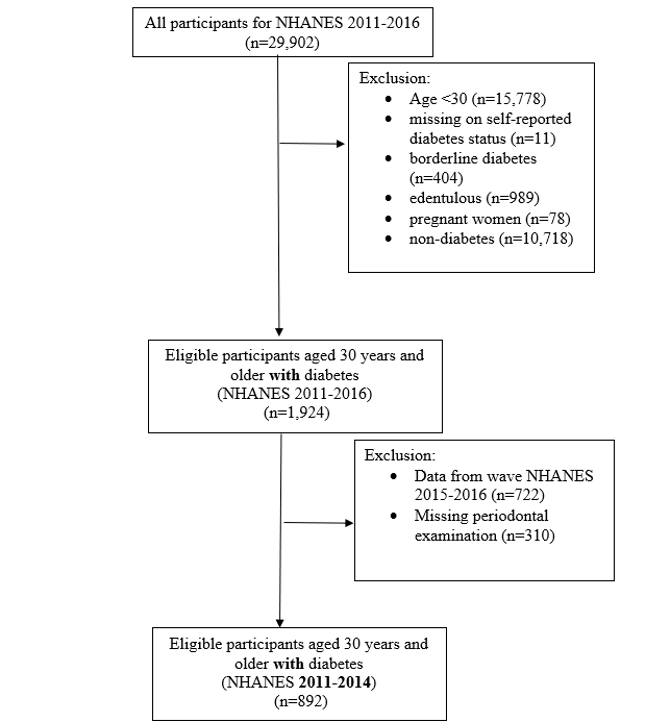

Supplement: Supplementary file 1 — Additional file 1: Supplemental Figure 1. Flow diagram for inclusion/exclusion to form the final analytical datasets. [file 12903_2023_3580_MOESM1_ESM.docx]
